# Unexpected Cardiac Asystole Caused by Vasovagal Reaction During Venipuncture: A Case Report

**DOI:** 10.5811/cpcem.50946

**Published:** 2026-04-07

**Authors:** Tomoki Nagano, Ryo Sakuma, Wataru Horiguchi, Soi Jeong, Takaki Tanamoto, Yumi Yokota, Matthew Fowler, Jin Kim

**Affiliations:** * Japanese National Physician Graduate Medical Education Program, United States Naval Hospital, Yokosuka, Japan; † United States Naval Hospital, Department of Internal Medicine, Yokosuka, Japan; ‡ United States Naval Hospital, Department of Emergency Medicine, Yokosuka, Japan

**Keywords:** vasovagal reaction, syncope, cardiac asystole, venipuncture, case report

## Abstract

**Introduction:**

The vasovagal reaction can lead to benign, self-limiting syncope triggered by stimuli such as pain or emotional stress. However, in rare and severe cases it may result in cardiac asystole. Previous episodes of vasovagal reactions could be a risk factor for cardiac asystole.

**Case Report:**

We present a 39-year-old male with a previous episode of vasovagal syncope who developed an unexpected 15-second episode of asystole during venipuncture, for which we performed immediate chest compressions. Further evaluations revealed no apparent underlying cause. The patient was subsequently diagnosed with transient asystole secondary to vasovagal reaction.

**Conclusion:**

While venipuncture is a common procedure in clinical practice, clinicians should be aware of the potential risk for cardiac asystole. Detailed medical history of previous episodes of vasovagal reactions could be helpful.

## INTRODUCTION

Venipuncture is the most common invasive procedure in hospitals and clinics. While typically associated with minor complications such as pain and bruising, it is regarded as safe and well-tolerated. However, a few studies have reported rare but serious complications, including sudden asystole secondary to vasovagal reaction.^1^–^6^ Herein, we present a case of unexpected asystole induced by venipuncture attributed to a severe vasovagal reaction. We also conducted a literature review to identify similar reported cases. We discuss the importance of detailed history-taking of previous vasovagal reactions as they may be potential red flags for predicting these events.

## CASE REPORT

**Image 1. A and B:**
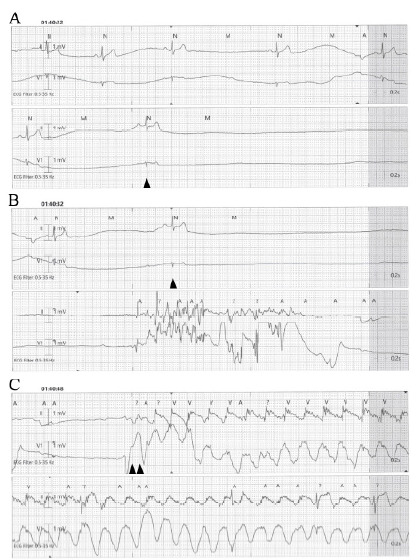
elemetry records immediately before the asystolic event (lead II and V1), with single arrowhead indicating the beginning of cardiac asystole. **C:** Double arrowheads indicate initiation of chest compressions.

A 39-year-old male with no significant past medical history presented with acute palpitations, which lasted for approximately 15 minutes. On arrival, his vital signs were as follows: heart rate, 89 beats per minute (bpm); respiratory rate, 12 breaths per minute; blood pressure, 113/74 millimeters of mercury; body temperature, 36.7 °Celsius; and oxygen saturation, 98% on room air. Initial 12-lead electrocardiogram (ECG) was unremarkable. The patient was placed on telemetry and a laboratory workup was initiated. During venipuncture, he complained of nausea and lightheadedness. The patient subsequently developed sinus bradycardia that eventually progressed to asystole, at which point he became unconscious. Asystole persisted for 15 seconds, and when no pulse was detected, chest compressions were initiated (Image 1). After 20 seconds of chest compression, he regained consciousness with return of spontaneous circulation. At that point, he was alert and oriented but appeared cool, clammy, and poorly perfused. He was, therefore, given one liter of normal saline with a pressure bag.

**Image 2:**
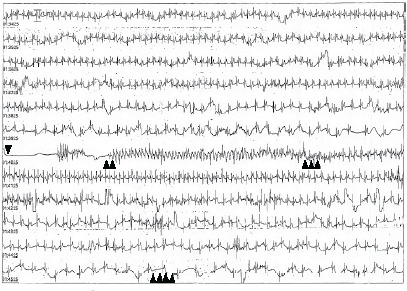
Monitoring record of syncopal episode. The single arrowhead indicates the beginning of cardiac asystole. Double arrowheads show the initiation of chest compressions, while triple arrowheads mark the return of consciousness and cessation of chest compressions. The quadruple arrowheads denote the administration of atropine.

Although his heart rate initially improved, it subsequently declined again to the mid-30s bpm. He was placed in the Trendelenburg position, and 0.5 mg of atropine was administered, which improved his heart rate to 100 bpm (Image 2). A repeat 12-lead ECG remained unremarkable. After regaining consciousness, the patient reported a previous syncopal episode during venipuncture that had resolved spontaneously, and he described experiencing intense anxiety after three failed venipuncture attempts during the episode. Further evaluation including lab tests and echocardiogram showed no identifiable causes. The patient did not report any orthostatic symptoms before or after the event. He was continuously monitored in the hospital for 24 hours with no events. The patient was diagnosed with transient asystole secondary to vasovagal reaction and was discharged without any symptoms.

*CPC-EM Capsule*

What do we already know about this clinical entity?

*Vasovagal reactions usually cause syncope but in rare cases can progress to severe bradycardia or even cardiac asystole*.

What makes this presentation of disease reportable?

*A healthy adult man developed transient cardiac asystole during venipuncture, despite no underlying cardiac abnormality*.

What is the major learning point?

*A history of prior vasovagal episodes may indicate increased susceptibility to asystole during minor procedures*.

How might this improve emergency medicine practice?

*Recognizing patients with prior vasovagal reactions allows proactive monitoring and early intervention during procedures*.

## DISCUSSION

This case suggests the following: 1) simple venipuncture can cause cardiac asystole secondary to a severe vasovagal reaction; and 2) previous episodes of vasovagal reactions could signify a risk. The differential diagnosis of transient asystole includes arrhythmogenic causes such as sinus node dysfunction, atrioventricular block, severe hyperkalemia-induced asystole, or acute myocardial infarction with conduction block. In this case, the patient’s baseline 12-lead ECG, lab findings, echocardiogram, and continuous 24-hour telemetry monitoring revealed no abnormalities, making these alternative diagnoses unlikely. Given his prior syncopal episode during venipuncture, the event was most consistent with transient asystole secondary to an exaggerated vasovagal reaction triggered by repeated venipuncture attempts. The primary pathophysiology of vasovagal reaction is thought to involve a cardioinhibitory response that results from either increased parasympathetic activation, a vasodepressor response due to inhibited sympathetic activity, or a combination of both mechanisms.^7^–^9^ Emotional stress or somatic pain can induce this cardioinhibitory response, which may result in profound bradycardia or hypotension.^10^ In severe cases, the vasovagal reaction has been reported to lead to asystole. In this case, the pain and emotional stress caused by multiple failed venipunctures were thought to have triggered a severe vasovagal reaction, leading to bradycardia followed by transient cardiac asystole.

To our knowledge, our case represents the seventh reported adult case of cardiac asystole caused by a venipuncture-related vasovagal reaction to be reported in the literature (Table). Interestingly, all six previously reported cases described a prior episode of vasovagal reaction. In this case, a detailed history-taking following the asystolic event revealed a previous, patient-reported episode of vasovagal syncope, suggesting a possible predisposition to exaggerated vagal responses. Individuals with recurrent vasovagal reactions are known to have a lower threshold for parasympathetic activation, predisposing them to bradycardia or even asystole under stress or painful stimuli.^10^ Since the patient was on telemetry during the venipuncture, the asystole was promptly detected, and timely intervention was started. While reports of similar cases are limited, previous episodes of vasovagal reactions could possibly represent a potential risk factor for cardiac arrest during these procedures, particularly in individuals with underlying autonomic hypersensitivity.

## CONCLUSION

Although venipuncture is a commonly performed procedure, clinical teams should be aware that it carries the rare potential of an exaggerated vasovagal reaction such as pronounced bradycardia and even asystole. History of previous venipuncture-related vasovagal symptoms should heighten awareness of potential adverse reactions. These patients can benefit from close observation and should trigger a low threshold for telemetry and escalation of care.

## Figures and Tables

**Table:** Previously published reports of cardiac asystole during venipuncture.

No.	Study	Age (years)	Sex	Time to ROSC	Emergent Intervention	Previous VVR
1	Reuben (1976)	44	F	2 minutes	Chest compression Epinephrine (1mg)	Yes (Syncope)
2	Lipton et al. (1993)	42	M	18 seconds	Chest compression Atropine (0.5mg)	Yes (Syncope)
3	Alijanian et al. (2001)	43	F	20 seconds	Chest compression	Yes (Syncope)
4	Wakita et al. (2006)	35	M	9 seconds	Atropine (0.25mg)	Yes (Syncope)
5	Matthews et al. (2021)	18	F	30 seconds	N/A	Yes (Syncope)
6	Shimoda et al. (2023)	38	M	18 seconds	N/A	Yes (Syncope)
7	Present case (2025)	39	M	35 seconds	Chest compression Atropine(0.5mg)	Yes (Syncope)

Abbreviations: *F*, female; *M*, male; *N/A*, not applicable; *mg*, milligram; *ROSC*, return of spontaneous circulation; *VVR*, vasovagal reaction.

## References

[1] Reuben T (1976). Cardiac arrest following routine venipuncture. JAMA.

[2] Lipton JD, Forstater AT (1993). Recurrent asystole associated with vasovagal reaction during venipuncture. J Emerg Med.

[3] Alijanian A, Bedrossian E, Feeney C, Devlin DH (2001). Asystole secondary to venipuncture: report of case. J Oral Maxillofac Surg.

[4] Wakita R, Ohno Y, Yamazaki S (2006). Vasovagal syncope with asystole associated with intravenous access. Oral Surg Oral Med Oral Pathol Oral Radiol.

[5] Matthews L, Kurukumbi M (2021). Reflex anoxic seizures induced by needle stick and successfully treated with intranasal midazolam. Cureus.

[6] Shimoda H, Yamauchi K, Takahashi T (2023). Transient asystole associated with vasovagal reflex in an oral surgery patient: a case report. SAGE Open Med Case Rep.

[7] Chen MY, Goldenberg IF, Milstein S (1989). Cardiac electrophysiologic and hemodynamic correlates of neurally mediated syncope. Am J Cardiol.

[8] Morillo CA, Eckberg DL, Ellenbogen KA (1997). Vagal and sympathetic mechanisms in patients with orthostatic vasovagal syncope. Circulation.

[9] Shim SH, Park SY, Moon SN (2014). Baseline heart rate variability in children and adolescents with vasovagal syncope. Korean J Pediatr.

[10] David LJ, Wouter W, Michele B (2018). Pathophysiology of the vasovagal response. Heart Rhythm.

